# Responses of Super Rice (*Oryza sativa* L.) to Different Planting Methods for Grain Yield and Nitrogen-Use Efficiency in the Single Cropping Season

**DOI:** 10.1371/journal.pone.0104950

**Published:** 2014-08-11

**Authors:** Song Chen, Danying Wang, Chunmei Xu, Chenglin Ji, Xiaoguo Zhang, Xia Zhao, Xiufu Zhang, Bhagirath Singh Chauhan

**Affiliations:** 1 China National Rice Research Institute, Chinese Academy of Agricultural Sciences, Hangzhou, Zhejiang, China; 2 Queensland Alliance for Agriculture and Food Innovation (QAAFI), University of Queensland, Toowoomba, Queensland, Australia; Zhejiang University, China

## Abstract

To break the yield ceiling of rice production, a super rice project was developed in 1996 to breed rice varieties with super high yield. A two-year experiment was conducted to evaluate yield and nitrogen (N)-use response of super rice to different planting methods in the single cropping season. A total of 17 rice varieties, including 13 super rice and four non-super checks (CK), were grown under three N levels [0 (N0), 150 (N150), and 225 (N225) kg ha^−1^] and two planting methods [transplanting (TP) and direct-seeding in wet conditions (WDS)]. Grain yield under WDS (7.69 t ha^−1^) was generally lower than TP (8.58 t ha^−1^). However, grain yield under different planting methods was affected by N rates as well as variety groups. In both years, there was no difference in grain yield between super and CK varieties at N150, irrespective of planting methods. However, grain yield difference was dramatic in japonica groups at N225, that is, there was an 11.3% and 14.1% average increase in super rice than in CK varieties in WDS and TP, respectively. This suggests that high N input contributes to narrowing the yield gap in super rice varieties, which also indicates that super rice was bred for high fertility conditions. In the japonica group, more N was accumulated in super rice than in CK at N225, but no difference was found between super and CK varieties at N0 and N150. Similar results were also found for N agronomic efficiency. The results suggest that super rice varieties have an advantage for N-use efficiency when high N is applied. The response of super rice was greater under TP than under WDS. The results suggest that the need to further improve agronomic and other management practices to achieve high yield and N-use efficiency for super rice varieties in WDS.

## Introduction

More than 90% of the world’s rice (*Oryza sativa* L.) is grown and consumed in Asia. In China, it is the staple food for about 60% of the population [1]. To meet the growing demand for food that will result from population growth and economic development in the next decade [2], great efforts should be made to breed new rice varieties with higher yield potential in order to enhance average farm yield [3], [4]. In the past decade, programs for super or ideotype rice breeding, aiming at dramatically increasing yield potential, have greatly progressed at the International Rice Research Institute (IRRI) and some countries, including Japan, Korea, and China [5]–[7]. In 1996, China established a nationwide mega-project on the development of super rice based on the ideotype concept [7]. A rice variety could be recognized as a super rice if it meets the yield target at two pilot sites in two successive years, or if it meets the goal of yield advantages over the control variety in regional yield trials. The criteria of super rice cultivars vary with both production area and subspecies type [7]. Up to 2012, 96 commercially released super rice varieties were grown on over a total of 80 million ha in China [8]. A number of super-high yield records of over 12 t ha^−1^ have been reported [4], [9], [10]. In addition, it was also reported that super hybrid rice varieties, such as Liangyoupeijiu and Xieyou 9308, produced 8–20% higher grain yield than check varieties, such as Shanyou 63 and Xieyou 63, in a similar environment [11], [12]. On the other hand, there was little increase in terms of large-scale rice production, indicating that the yield potential of newly released super rice varieties has not been fully realized, mainly because of unreasonable agronomic practices. Thus, it is imperative to understand yield responses of super rice to various agronomic practices, such as N rates or planting methods.

Traditionally, rice is cultivated using the transplanting method, consisting of raising nurseries, uprooting and picking seedlings, and transplanting [13], which cost labor and energy [14], [15]. Paradoxically, labor availability is limited in China because an increasing number of young farmers have left for jobs in the cities [16]. Direct-seeded rice is an alternative production system that can help reduce labor costs [17]. Therefore, the planting pattern in the past two decades is being gradually and partially replaced by direct seeding in many countries [18], [19]. This is also the case in China; the rice area for wet direct seeding (WDS) is rapidly increasing [13]. Previous studies suggest that WDS could be adopted on a large area in the Yangtze River Basin [20]. Furthermore, WDS cultivation provides rice with a completely different growth condition at the seedling stage than cultivation by transplanting does. Studies on rice under WDS indicate favorable changes for high yield formation in comparison with transplanted rice [21]–[23]. These changes include earlier seedling emergence [21], stronger root activity, higher seed setting rate [22], and greater biomass production at the early stage [22], [23]. On the other hand, a significant improvement in grain yield has not been achieved in WDS systems because of unstable seedling establishment, sensitivity to lodging, shorter growth period, and severe competition with weeds [17], [20]. Generally, most super rice varieties currently planted in China are developed by transplanting (TP) cultivation. Therefore, further research is needed to evaluate the different genotypic responses in growth and yield of super rice to planting methods.

Nitrogen (N) is the most important yield-limiting nutrient for rice [24]. Increased rice production is largely attributed to the increased use of N fertilizer. The amount of N uptake needed to produce one ton of rough rice is 15–17 kg N for an average yield of 5–6 t ha^−1^ [24]; it is 19 kg for high-yielding rice [25]. For super rice varieties, the amount of N uptake was more than 18 kg N t^−1^ of grain yield, and, in some cases, even reached as high as 20 kg N t^−1^ of grain yield [9], [12], [26]–[28]. Some super rice varieties, such as Liangyoupeijiu, were bred for high fertility conditions with high N application, achieving the yield of 10.98 t ha^−1^ with an application of 234 kg N ha^−1^ [9]. An earlier study also indicated that japonica super rice in north China reached a very high yield (11 t ha^−1^) with the application of more than 247.5 kg N ha^−1^ [29]. In China, 150 kg N ha^−1^ applied at key growth stages is recommended for traditional varieties in TP systems [30]. Therefore, the abundant use of N fertilizer on super rice on a large scale would generally accelerate non-point source pollution. In addition, it was also reported that TP has a greater N-use efficiency than WDS in six non-super indica varieties [31]. However, beyond the yield output, as well as for relative physiological or morphological traits, the study of the effect of planting methods on N-use efficiency in high-yielding varieties is still limited.

In this study, a two-year field research was conducted to compare the yield performance of super rice varieties with three N levels (0, 150, and 225 kg ha^−1^) and two planting methods (TP and WDS). The objectives of this study are to (1) evaluate the grain yield performance of super rice in different planting systems, and (2) evaluate the N-use efficiency of super rice varieties, switching from TP to WDS.

## Materials and Methods

### Experimental design and crop management

Experiments were conducted in 2011 and 2012 at the experimental farm of the China National Rice Research Institute, Fuyang County (28′09′N, 113′37′E, 43 m a.s.l.), Zhejiang Province, China. The soil was clay with the following properties: pH 6.0, 22.3 g kg^−1^ organic matter, 197 mg kg^−1^ alkali-hydrolyzale N, 102.8 mg kg^−1^ available P, and 205 mg kg^−1^ available K. The soil test was based on samples taken from the upper 20 cm of the soil.

Treatments were arranged in a split-split-plot design with planting method in the main plots, N rate in split-plots, and variety in split-split plots. The experiment was replicated three times and the sub-sub plot size was 30 m^2^.

A total of 17 and 15 varieties in the single rice cropping system were used in 2011 and 2012, respectively. Four groups of varieties were chosen based on the subspecies and super yield: high-yielding indica hybrid varieties, high-yielding japonica inbred varieties, and indica and japonica check varieties. These varieties have been widely grown by farmers in the Yangtze River Basin in China. Details on the varieties are given in Table 1.

**Table 1:** Details on the varieties used in the experiment.

Variety	Type	Year of release	Growth period (d)	Plant height (cm)
II-you838	aHybrid Indica -CK	1995	140–145	98–102
Shanyou63	Hybrid Indica -CK	1984	138.0	105–110
II-Youhang1	Hybrid Indica	2005	135.8	127.5
II-Youhang2	Hybrid Indica	2007	134.5	129.9
Fengliangyou6	Hybrid Indica	2008	145.0	121.8
Neiliangyou6	Hybrid Indica	2007	154.4	110.4
Liangyoupei9	Hybrid Indica	2001	150.0	110–120
Peiliangyou3076	Hybrid Indica	2006	135.7	119.1
Yangliangyou6	Hybrid Indica	2005	134.1	120.6
Zhongzheyou1	Hybrid Indica	2004	138.0	125.0
Xiushui09	bJaponica-CK	2005	159.0	92.5
Xiushui63	Japonica-CK	1997	150–154	95–100
Huaidao11	Japonica	2008	156.0	103.9
Huaidao9	Japonica	2006	152.0	100.0
Ningjing1	Japonica	2004	145.0	93.1
Ningjing3	Japonica	2008	159.0	98.6
Yangjing4038	Japonica	2008	135.0	106.0

a The hybrid Indica rice were hybrid varieties developed using the three-line method.

b The Japonica rice were inbred varieties.

The information of varieties was obtained from the China Rice Data Center (http://www.ricedata.cn/index.htm).

We confirm that our study did not involve endangered or protected species. No specific permissions were required for using the locations and varieties. The seeds of all varieties used in the study are commercially available.

Two planting methods were included: manual wet direct seeding (WDS) and transplanting (TP). For TP, pre-germinated seeds were sown on a seedbed. Thirty- and 25-d-old seedlings were transplanted on 20 June 2011 and 25 June 2012, respectively. Two seedlings were transplanted at a spacing of 30 cm×16.7 cm. In WDS, pre-germinated seeds (about 3–4 seeds per hill) were placed manually on the surface of puddled soil, with a spacing of 30×16.7 cm. To maintain similar plant density, excess seedlings were removed after emergence (about 10–15 days after seeding).

Three N treatments were included: 0 (N0), 150 (N150), and 225 (N225) kg N ha^−1^. For N150, 75, 45, and 30 kg N ha^−1^ were applied at basal, mid-tillering, and panicle initiation, respectively. For N225, 113, 67, and 45 kg N ha^−1^ were applied at basal, mid-tillering, and panicle initiation, respectively. Phosphorus (60 kg P_2_O_5_ ha^−1^) was applied and incorporated in all plots 1 day before TP or WDS. Potassium (150 kg K_2_O ha^−1^) was applied in two equal splits at basal and panicle initiation in both establishment methods.

Crop management followed the standard cultural practices. Weeds in the field were well controlled using intensive hand weedings. The experimental field was kept flooded from the day of transplanting until 10 days before maturity in both planting methods. Insects were intensively controlled by chemicals to avoid biomass and yield loss.

### Sampling and measurements

At maturity, 12 hills were sampled diagonally from a 5-m^2^ harvest area and were separated into panicle and straw to determine the dry weight. Dry weight was determined after oven-drying the samples at 70°C to the constant weight. Grain yield was determined from a 5-m^2^ area in each plot and adjusted to the standard moisture content of 14%. The N concentrations of straw and panicles were determined by micro-Kjeldahl digestion, distillation, and titration [32]. N accumulation (panicle or straw) resulted from dry weight and N concentration (panicle or straw). Total N accumulation was calculated from the panicles and straw.

Nitrogen agronomic efficiency (NAE), nitrogen physiological efficiency (NPE), and nitrogen-recovery efficiency (NRE) were calculated as:
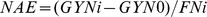





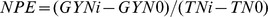





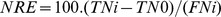



In the equations, *GYNi* is the grain yield from the plots that received N fertilizer; *GYN0* is the grain yield in the zero-N (control) plots; *FNi* is the amount of N fertilizer applied; *TNi* is the total above plant N accumulation in the plots that received N fertilizer; and *TN0* is the total above plant N accumulation in zero-N (control) plots.

### Statistical analyses

Data generated from the experiments were subjected to statistical software SAS 8.0 for Windows separately for 2011 and 2012. A three-way analysis of variance (ANOVA) was conducted for all of the abovementioned parameters from three replicates at harvest, with the following effects: variety group, nitrogen rates, planting methods, variety group×N rates, variety group×planting methods, planting methods×N rates, and variety group×N rates×planting methods. Yield comparisons were made among the combination of N rates and planting methods for each variety group using Tukey’s HSD; a *p* value <0.05 was considered significantly different.

## Results

ANOVA results for grain yield, N accumulation, NAE, NRE, and NPE are shown in Table 2. The year effect was significant; therefore, the results are presented and discussed separately for years.

**Table 2:** Analysis of variance for nitrogen (N), planting method, and variety group on grain yield, total N accumulation, NAE, NPE, and NRE in 2011 and 2012.

Factor	Grain yield (t ha^−1^)		Total N accumulation (kg ha^−1^)		NAE (kg grain kg^−1^ N)		NPE (kg grain kg^−1^ N)		NRE (%)	
	2011	2012	2011	2012	2011	2012	2011	2012	2011	2012
Nitrogen (N)	**	**	**	**	**	**	**	**	**	**
Planting method (P)	**	**	**	**	ns	ns	*	*	ns	ns
Variety group (V)	**	**	*	ns	**	**	**	**	*	*
P*N	ns	*	ns	ns	*	*	*	*	ns	ns
V*N	ns	ns	**	*	*	*	**	**	*	*
V*P	**	*	*	*	*	*	ns	ns	ns	ns
V*P*N	**	*	*	*	*	*	*	*	**	**

Abbreviations: *means the p value at <0.01; **means the p value at <0.001; ns, nonsignificant; NAE, N agronomic efficiency; NPE, N physiological efficiency; NRE, N recovery efficiency.

### Grain yield

In 2011, grain yield at N150 ranged from 7.5 to 9.3 t ha^−1^ in WDS and 8.2 to 10.3 t ha^−1^ in TP, whereas, the corresponding values at N225 were 6.5–9.5 t ha^−1^ and 7.3–10.9 t ha^−1^, respectively (Table 3). In 2012, grain yield at N150 ranged from 7.5 to 9.9 t ha^−1^ in WDS and 7.9 to 10.3 t ha^−1^ in TP, whereas, the corresponding values at N225 were 6.1–9.1 t ha^−1^ and 7.4–10.9 t ha^−1^, respectively (Table 4). The interaction of N rates, planting methods, and variety groups was significant for grain yield (Table 2). In general, grain yield under WDS was lower than TP–9.1% and 10.6% lower in 2011 and 2012, respectively. However, the differences varied with N rates and planting methods. At N225, the average grain yield for super japonica, super indica, and japonica CK varieties in 2011 under TP were 9.03, 8.90, and 10.36 t ha^−1^. These were 20.9%, 11.9%, and 15.6% higher than those under WDS, respectively. However, no difference was found between TP and WDS for indica CK varieties. Similar results were also found in 2012. In 2011, at N150, yield increases of 0.85 and 1.18 t ha^−1^ were obtained under TP than under WDS for super varieties japonica and indica, respectively; there was no difference found for CK varieties. In 2012, yield increase in transplanted rice was found only for super japonica varieties, and no difference was found for the rest of the variety groups.

**Table 3:** Effect of nitrogen (N) rates [N0, N150, and N225 are 0, 150, and 225 kg N ha^−1^, respectively] and planting methods (transplanting, TP and wet direct seeding, WDS) on grain yield in 2011.

Variety	N0		N150		N225	
	WDS	TP	WDS	TP	WDS	TP
CK (Indica)						
II-you838	7.0±0.2	7.2±0.4	8.1±0.8	9.0±0.5	7.8±0.6	7.7±0.2
Shanyou63	7.5±0.6	7.9±0.5	8.5±0.6	8.5±0.4	8.1±0.3	8.5±0.6
Average	7.3b	7.6b	8.3a	8.7a	8.0ab	8.1ab
High yielding varieties (Indica hybrid)						
II-Youhang1	5.4±0.2	7.2±0.3	8.0±0.4	9.9±0.9	7.4±0.7	9.1±0.2
II-Youhang2	5.5±0.4	6.2±0.3	8.1±0.4	8.7±0.8	8.0±0.8	10.5±0.3
Fengliangyou6	5.9±0.6	6.4±0.5	7.9±0.4	8.5±0.6	7.9±0.5	8.1±0.2
Neiliangyou6	6.3±0.3	8.2±0.5	8.0±0.5	10.1±0.4	8.3±0.7	10.3±0.6
Liangyoupei9	5.7±0.2	7.3±0.5	8.3±0.4	8.4±0.6	6.5±0.5	10.2±0.3
Peiliangyou3076	5.6±0.3	6.5±0.4	8.4±0.8	8.4±0.4	6.9±0.6	7.3±0.7
Yangliangyou6	5.9±0.3	7.1±0.2	7.5±0.3	8.3±0.4	6.6±0.3	7.6±0.3
Zhongzheyou 1	6.5±0.3	7.0±0.4	8.6±0.3	9.3±0.9	8.1±0.3	9.1±0.5
Average	5.9d	7.0c	8.1b	9.0a	7.5bc	9.0a
CK (Japonica)						
Xiushui09	7.0±0.7	7.9±0.7	9.3±0.4	9.9±0.7	8.3±0.6	9.8±0.9
Xiushui63	6.1±0.2	6.8±0.5	8.2±0.7	8.2±0.6	7.6±0.2	8.0±0.3
Average	6.6c	7.4b	8.8a	9.1a	8.0b	8.9a
High yielding varieties (Japonica)						
Huaidao11	6.1±0.4	8.5±0.4	7.9±0.7	9.7±0.5	8.6±0.4	10.4±0.8
Huaidao9	6.6±0.4	7.8±0.6	9.3±0.5	10.3±0.7	9.5±0.6	10.0±0.4
Ningjing1	6.3±0.5	7.9±0.2	8.2±0.7	9.4±0.9	9.4±0.4	10.2±0.4
Ningjing3	6.6±0.5	7.6±0.6	8.5±0.8	9.9±0.4	8.6±0.7	10.3±0.3
Yangjing4038	7.2±0.2	7.6±0.5	8.2±0.6	8.6±0.7	8.7±0.7	10.9±1
Average	6.6d	7.9c	8.4bc	9.6ab	9.0b	10.4a

Yields of rice varieties were expressed as Mean ± SE (with 3 replications). Average means in rows followed by different letters were significantly different at *p*<0.05 (Tukey’s HSD).

**Table 4:** Effect of nitrogen (N) rates [N0, N150, and N225 are 0, 150, and 225 kg N ha^−1^, respectively] and planting methods (transplanting, TP and wet direct seeding, WDS) on grain yield in 2012.

Variety	N0		N150		N225	
	WDS	TP	WDS	TP	WDS	TP
CK (Indica)						
II-you838	7.5±0.4	7.5±0.6	8.1±0.7	9.5±0.9	8.2±0.8	8.0±0.5
Shanyou63	7.3±0.6	7.8±0.7	8.3±0.3	8.1±0.6	8.7±0.2	8.8±0.3
Average	7.4b	7.7b	8.2ab	8.8a	8.4a	8.4a
High yielding varieties (Indica hybrid)						
II-Youhang1	5.0±0.4	6.9±0.6	8.5±0.4	9.9±0.8	7.7±0.6	8.5±0.4
II-Youhang2	5.8±0.2	6.6±0.3	7.5±0.2	8.5±0.4	7.6±0.6	10.2±0.4
Neiliangyou6	6.2±0.6	7.8±0.5	7.9±0.7	9.6±0.7	8.5±0.7	9.8±0.4
Liangyoupei9	5.4±0.3	7.6±0.4	8.2±0.7	7.9±0.6	6.7±0.4	9.7±0.6
Peiliangyou3076	5.6±0.2	6.8±0.1	8.7±0.2	8.4±0.8	7.4±0.3	7.4±0.2
Zhongzheyou1	6.6±0.4	7.2±0.5	8.5±0.7	9.1±0.9	8.0±0.2	9.3±0.5
Average	5.8c	7.2b	8.2ab	8.9a	7.7b	9.1a
CK (Japonica)						
Xiushui09	7.8±0.8	9.9±0.7	10.2±0.6	8.0±0.7	9.5±0.7	7.8±0.8
Xiushui63	6.7±0.4	7.7±0.8	8.7±0.4	7.2±0.6	8.0±0.4	6.7±0.4
Average	6.5c	7.2b	8.8ab	9.5a	7.6b	8.7ab
High yielding varieties (Japonica)						
Huaidao11	8.6±0.4	7.8±0.4	9.2±0.7	9.1±0.4	10.9±0.3	8.6±0.4
Huaidao9	7.3±0.2	9.2±0.4	10.3±0.5	8.8±0.5	10.5±0.9	7.3±0.2
Ningjing1	8.2±0.5	8±0.3	9.5±0.3	9.0±0.8	10.7±0.4	8.2±0.5
Ningjing3	7.5±0.5	8.2±0.5	9.7±0.4	8.8±0.6	9.9±0.8	7.5±0.5
Yangjing4038	7.5±0.6	8.7±0.5	8.8±0.7	8.1±0.5	10.8±0.7	7.5±0.6
Average	6.6c	7.8b	8.4b	9.5ab	8.8b	10.6a

Yields of rice varieties were expressed as Mean ± SE (with 3 replications). Average means in rows followed by different letters were significantly different at *p*<0.05 (Tukey’s HSD).

These results indicate that super rice has a greater yield potential at high N rates in the TP system. The optimum N rate for wet direct-seeded rice was 150 kg ha^−1^ in the current condition. In both years, grain yield at N150 and N225 was similar, except for super indica and japonica CK varieties under the WDS system, in which grain yield was 8.5% and 10.1% higher at N150 than at N225, respectively. However, yield at N150 and N225 were always greater than that at N0. At N0, the average grain yield of super varieties was relatively lower than that of CK varieties. For indica varieties under WDS, average grain yield of the checks (7.25 t ha^−1^ and 7.69 t ha^−1^) was 23.9% and 27.6% higher than that of super varieties in 2011 and 2012, respectively. In both years, irrespective of planting method, no difference was found in grain yield between super and CK varieties at N150. At N225, however, grain yield difference was dramatic for the japonica group; there was an 11.3% and 14.1% average increase in grain yield for super varieties under WDS and TP, respectively, in comparison with the checks. In both years, for indica, the grain yield of super varieties (9.1 t ha^−1^) was 9.2% higher than CK varieties (7.5 t ha^−1^) under the TP system, but 8.4% lower under the WDS system. Such cases of yield decline for super indica varieties at N225 under WDS might lead to the negative effect of super varieties (e.g., Liangyoupei9 and Peiliangyou3076). The yield decline was generally due to the excessive growth of plants and/or lodging during panicle initiation and/or grain filling (visual observations). The results imply a potential risk to super varieties applied with high N doses in the WDS system.

### Total nitrogen accumulation

**Figure 1:**
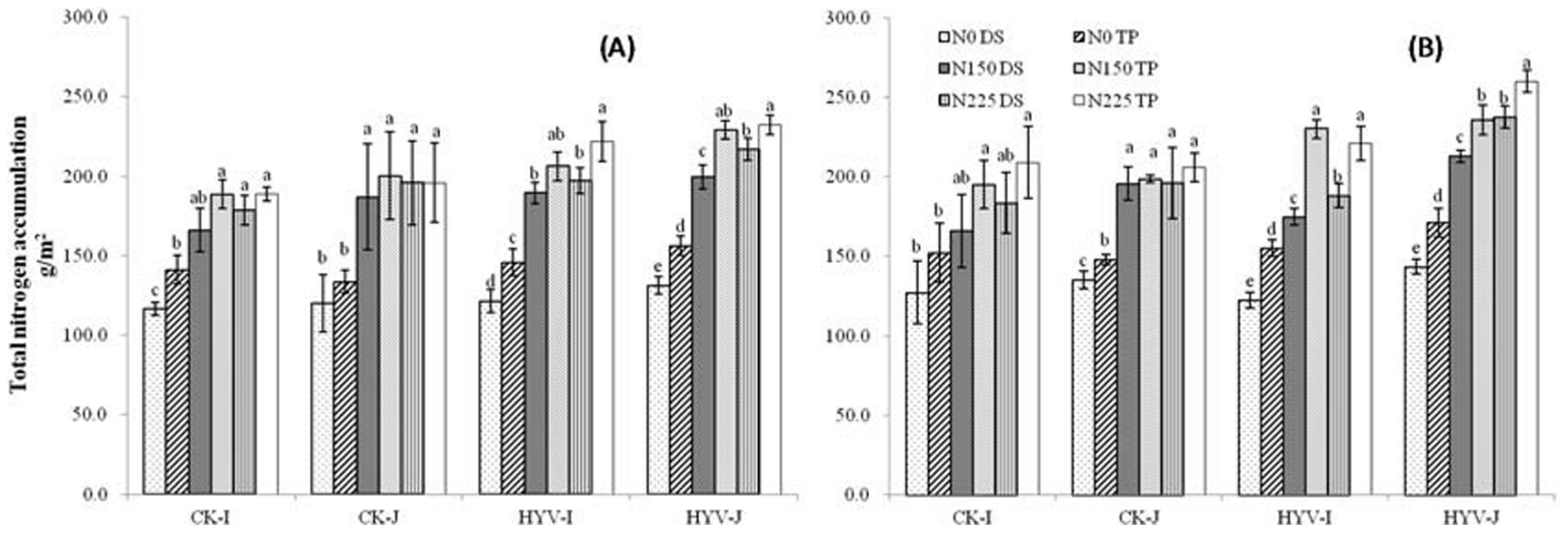
Effect of nitrogen (N) rates and planting methods on total nitrogen accumulation in above biomass in 2011 (A) and 2012 (B). WDS, wet direct-seeded rice; TP, transplanting rice; N0, 0 kg N ha^−1^; N150, 150 kg N ha^−1^; N225, 225 kg N ha^−1^. The vertical bars stands for standard error of means. Average means followed by different letters were significantly different at *p*<0.05 (Tukey’s HSD) in the same variety group.

N×planting method×variety group interaction was significant. However, N×planting method interaction was not significant (Table 2), suggesting the consistent effect of planting methods on total nitrogen accumulation (TNA) at different N rates (Fig. 1). In 2011, average TNA values under WDS across N rates were 153.9, 169.4, and 182.6 g m^−2^ for indica CK, super indica, and super japonica varieties, respectively, which were 14.6%, 12.0%, and 11.8% lower than those under TP, respectively. However, no difference was found for japonica CK. Similar results were found in 2012, except for japonica CK at N0, in which TNA values increased under WDS than under TP.

Differences among variety groups varied with N rates and planting methods. In 2011, no difference was found between super and CK varieties for the indica group, except for plants grown at N150 in the TP system, in which TNA was 17.9% higher in super rice than in CK varieties. In japonica, TNA in the TP system was 15.9% and 18.6% higher in super rice than in CK varieties at N0 and N150, respectively. However, no difference was found in the WDS system. A significant increase in TNA between super and CK varieties was found at N225 in both planting methods–21.1% and 26.3% greater in super rice than in CK varieties. In 2012, similar results were found for both japonica and indica groups, except at N0 under TP, in which no difference was detected between super and CK varieties.

### Nitrogen agronomic efficiency, N-recovery efficiency, and N physiological efficiency

**Figure 2:**
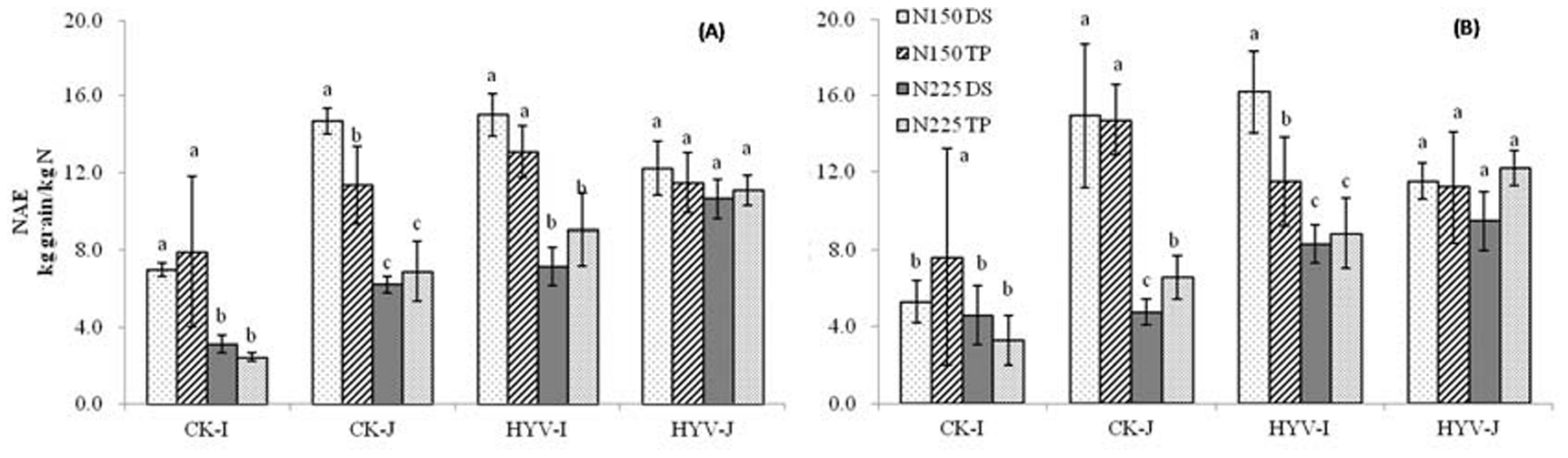
Effect of nitrogen (N) rates and planting methods on N agronomic efficiency (NAE) in 2011 (A) and 2012 (B). WDS, wet direct-seeded rice; TP, transplanting rice; N0, 0 kg N ha^−1^; N150, 150 kg N ha^−1^; N225, 225 kg N ha^−1^. The vertical bars stands for standard error of means. Average means followed by different letters were significantly different at *p*<0.05 (Tukey’s HSD) in the same variety group.

In both years, irrespective of N rate and variety group, no difference was found in NAE between the TP and WDS systems, except for the super indica varieties and japonica CK varieties at N150 in 2011, in which NAE was greater in WDS than in TP (Fig. 2). In the indica group in 2011, NAE was greater (66–270%) in super rice (11.1 kg grain kg^−1^ N on average) than in CK varieties (5.1 kg grain kg^−1^ N on average). However, the difference in japonica varieties varied with N rate. No difference was found for NAE at N150, whereas super rice varieties (10.9 kg grain kg^−1^ N on average) had greater NAE than CK varieties (6.5 kg grain kg^−1^ N on average) at N225–71% and 61% greater under WDS and TP, respectively. In 2012, irrespective of planting method and variety group, the difference between super and CK varieties was not significant at N150, except for indica varieties under WDS, in which the average NAE in super varieties was 206% greater than that in CK varieties. Regardless of planting method and variety group, super varieties had greater NAE values than CK varieties at N225. A higher NAE in super varieties at N150 and N225 for indica and only at N225 for japonica suggests that indica super varieties might be more sensitive to N than japonica super varieties.

**Figure 3:**
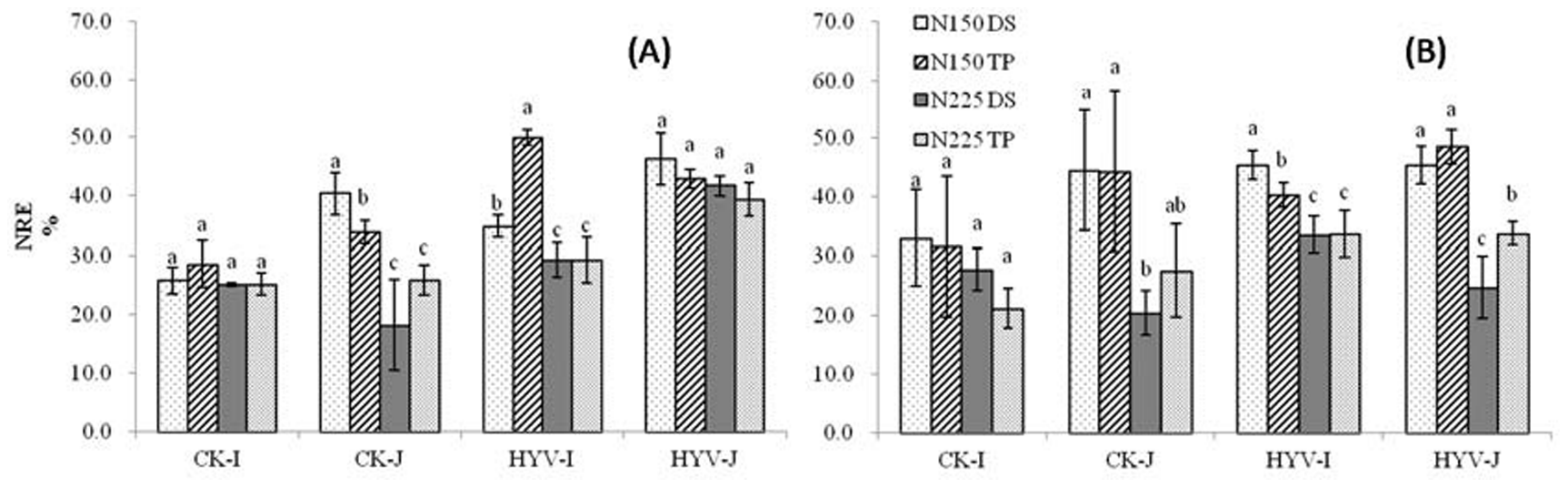
Effect of nitrogen (N) rates and planting methods on N recovery efficiency (NRE) in 2011 (A) and 2012 (B). WDS, wet direct-seeded rice; TP, transplanting rice; N0, 0 kg N ha^−1^; N150, 150 kg N ha^−1^; N225, 225 kg N ha^−1^. The vertical bars stands for standard error of means. Average means followed by different letters were significantly different at *p*<0.05 (Tukey’s HSD) in the same variety group.

The difference in NRE was not significant between TP and WDS, except for the japonica CK variety at N225, in which the NRE was 42% and 36% greater in TP than in WDS (Fig. 3). In both years, irrespective of N rate and planting method, no difference was found for NRE between super and CK varieties, except for japonica at N225 in 2011, in which the NRE was 130% and 53% greater in the super rice varieties than in the CK varieties under WDS and TP, respectively (Fig. 3).

**Figure 4:**
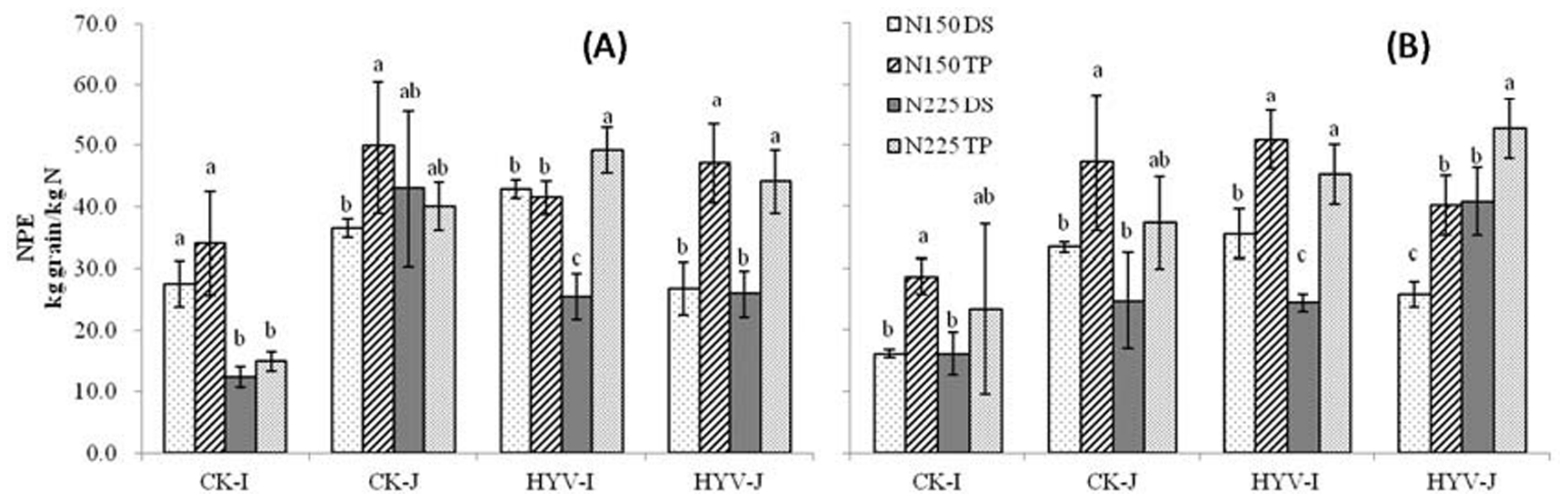
Effect of nitrogen (N) rates and planting methods on N physiological efficiency (NPE) in 2011 (A) and 2012 (B). WDS, wet direct-seeded rice; TP, transplanting rice; N0, 0 kg N ha^−1^; N150, 150 kg N ha^−1^; N225, 225 kg N ha^−1^. The vertical bars stands for standard error of means. Average means followed by different letters were significantly different at *p*<0.05 (Tukey’s HSD) in the same variety group.

In both years, irrespective of N rate and variety group, plants under TP had generally greater NPE than those under WDS, except for super indica varieties at N150 and japonica varieties at N225 in 2011, in which no difference was found between TP and WDS (Fig. 4). The average NPE in the TP system was 43 and 42 kg grain kg^−1^ N in 2011 and 2012, respectively, which were 33% and 55% higher than in the WDS system. Among varieties, NPE was generally higher in super rice than in CK varieties for the indica group, but it was similar for the japonica group. In both years, the average NPE of super indica varieties was 39 kg grain kg^−1^ N, which was 82% higher than that of CK varieties.

## Discussion

### Grain yield response to planting method

In current rice production systems with high economic outputs, seedling establishment has become more important than ever. Direct seeding methods have been suggested to replace the traditional transplanting method because of their advantage in saving labor without yield loss for high-yielding rice varieties [20]. In general, rice yield under direct seeding in farmers’ fields is lower than under the transplanting method [19]. However, there is a genotypic and environmental interaction for the yield of direct-seeded rice. No distinct difference in grain yield was found between direct seeding and transplanting under flooded conditions [20], [33]. A previous study reported that direct-seeded rice had superior grain yield than transplanted rice when short-duration varieties were used, but had equal or lower grain yield when medium- and long-duration varieties were used [34].

In our study, grain yield under WDS (7.69 t ha^−1^) was generally lower (9.9%) than under TP (8.58 t ha^−1^). However, the effect of planting methods on grain yield varied with N rate and variety group. No difference was found between TP and WDS at N150 in all the variety groups, except for the super japonica varieties. These results indicate that the optimum N rate for rice under WDS is 150 kg ha^−1^ in current conditions. In addition, the reduced growth period, ranged from 6–12 days across nitrogen levels and years, was also observed in the WDS system (data not shown), which might have reduced the photosynthetic duration during grain filling, reduced the biomass accumulation, and finally resulted in the yield decline [33]. Interestingly, Liangyoupei9 and Peiliangyou2046 had greater grain yield in WDS than in TP, indicating the possibility of breeding varieties suitable for direct seeding at optimum N rates.

### Grain yield response to super rice varieties

A total of 13 and 11 super high-yielding rice varieties (hybrid indica and inbred japonica) were used in 2011 and 2012, respectively. These varieties have been approved as super rice and widely spread in farmers’ fields in China (www.moa.gov.cn). Many field experiments have shown that super rice can achieve a grain yield of around 12 t ha^−1^ [7], [9], [10]; however, the yield output of farmers’ fields is still arguable. One of the important results of our study is that 10.9 t ha^−1^ was the highest yield observed under the environment at Fuyang, China. The high-yielding varieties did not meet the grain yield criteria for super rice, which was a minimum of 11.7 t ha^−1^ for a single season of indica/japonica rice in the Yangtze River Basin. This could partly be the effect of the environment on yield behavior and indicates a need for further work on improving yield performance of super rice varieties through agronomic and other management practices. On the other hand, relative grain yield was still superior for super rice varieties than for CK varieties. Current results show that the effect of super rice varieties on grain yield varied with the variety group. In both years, no difference was found for grain yield between super rice and CK varieties at N150 regardless of planting method. However, grain yield difference was dramatic in the japonica group at N225. Averaged over years, super rice varieties had 11.3% and 14.1% higher yield than CK varieties in the WDS and TP systems, respectively. The results suggest that high N input could contribute to narrowing the yield gap in super rice varieties and also provide evidence that super rice was bred for high fertility conditions.

### Nitrogen-use efficiency traits of super rice

Nitrogen is one of the most active elements for rice growth and yield performance. To achieve improved yield over traditional varieties, super rice varieties are generally bred in fertile conditions through transplanting [9], [26], [27]. During the last decade, the effect of N application on the NUE of super rice has been well-studied in the TP system; however, contradictory results still exist. In a recent study, the NUE of super japonica rice is greater at 246 kg N ha^−1^ than at 214 kg N ha^−1^ [35]. Another study compared the NPE and NAE of super rice Shennong265 and CK Liaojing294 at two N levels (150 and 250 kg ha^−1^) and reported that the NPE and NAE values of the super rice variety were greater than that of the CK variety at higher N application and lesser at lower N application [36]. Moreover, the physiological parameters affecting NUE also changes when the planting method shifts from TP to WDS. In general, panicle density of direct-seeded rice was greater than that of transplanted rice, but spikelet number per panicle was reduced because of the internal compensation [37]. It was also reported that the lower tissue N concentration during spikelet differentiation in direct-seeded rice may limit spikelet numbers compared with transplanted rice [21], [37]. In our study, more N was accumulated in super rice than in the check at N225 in japonica, but no difference was found between super and CK varieties at N0 and N150, indicating that the yield increase of japonica super rice resulted from high N inputs. Similar results were also found for NAE. These results suggest that super rice varieties have an advantage at NUE when high N is applied.
